# Decadal stability of radiocesium inventories and soil to tree transfer in forests affected by the Fukushima nuclear accident

**DOI:** 10.1038/s41598-025-34898-0

**Published:** 2026-01-07

**Authors:** Wataru Sakashita, Naohiro Imamura, Shinta Ohashi, Masabumi Komatsu, Masatake G. Araki, Satoshi Saito, Takuya Kajimoto, Shoji Hashimoto, Takuya Manaka, Tadashi Sakata, Yoshimi Ohmae, Satoru Miura, Yoshiki Shinomiya

**Affiliations:** 1 Center for Forest Restoration and Radioecology, Forestry and Forest Products Research Institute (FFPRI), 1 Matsunosato, Tsukuba, Ibaraki 305-8687 Japan; 2 Department of Forest Soils, FFPRI, 1 Matsunosato, Tsukuba, Ibaraki 305-8687 Japan; 3 Hokkaido Research Center, FFPRI, 7 Hitsujigaoka, Toyohira-ku, Sapporo, Hokkaido 062-8516 Japan; 4 Department of Wood Properties and Processing, FFPRI, 1 Matsunosato, Tsukuba, Ibaraki 305-8687 Japan; 5 Research Planning Department, FFPRI, 1 Matsunosato, Tsukuba, Ibaraki 305-8687 Japan; 6 Department of Mushroom Science and Forest Microbiology, FFPRI, 1 Matsunosato, Tsukuba, Ibaraki 305-8687 Japan; 7 Department of Plant Ecology, FFPRI, 1 Matsunosato, Tsukuba, Ibaraki 305-8687 Japan; 8 Sado Island Center for Ecological Sustainability, Niigata University, 94-2 Koda, Sado, Niigata 952-2206 Japan

**Keywords:** Radiocesium, Forest, Aggregated transfer factor, Quasi-equilibrium state, Fukushima Daiichi Nuclear Power Plant accident, Ecology, Ecology, Environmental sciences

## Abstract

Understanding the long-term dynamics of radiocesium (^137^Cs) in forests contaminated by the Fukushima nuclear accident requires determining when these dynamics reach a quasi-equilibrium state, in which the flux of ^137^Cs between soil and trees becomes approximately balanced. In this study, we analyzed time-series variations from 2011 to 2020 in the total ^137^Cs inventory and ^137^Cs distribution in aboveground compartments (needles/leaves, branches, bark, sapwood, and heartwood). Time-series analysis using a dynamic linear model indicated that the decay-corrected total ^137^Cs inventory (as of September 1, 2020) and its distribution in the aboveground compartments remained stable from 2017 onward, suggesting that a quasi-equilibrium state had been reached. Given this stability in both inventory and distribution, the aggregated transfer factor (*T*_*ag*_)—defined as the ^137^Cs activity concentration in aboveground tree tissues divided by the total ^137^Cs inventory in soil—measured approximately 6 years after the Fukushima nuclear accident can be considered representative of the long-term transfer of ^137^Cs from forest soil to trees. These findings on the stability of ^137^Cs in forests provide valuable insights for validating the accuracy of models that predict long-term ^137^Cs activity concentration in stem wood.

**Supplementary Information:**

The online version contains supplementary material available at 10.1038/s41598-025-34898-0.

## Introduction

After the Fukushima Daiichi Nuclear Power Plant (FDNPP) accident, numerous studies have examined the dynamics and redistribution of radiocesium (^137^Cs) within the forest ecosystem^1–3^. In evergreen coniferous forests, more than 60% of the ^137^Cs released from the FDNPP accident was initially intercepted by the canopy^4^. In contrast, in deciduous broad-leaved forests, canopy interception was limited because the trees were leafless at the time of the accident^5^. The intercepted ^137^Cs was subsequently transferred from the canopy to the forest floor through hydrological (throughfall and stemflow) and biological (litterfall) processes^6–9^. ^137^Cs that reached the forest floor migrated downward from the organic horizon to the surface mineral soil horizon over time^10–12^. At present, most of the ^137^Cs is retained in the surface mineral soil horizon due to fixation by clay minerals^13–15^. In recent years, observational studies have investigated the distribution and dynamics of ^137^Cs after the *early phase*, defined as the period of rapid redistribution of the initial deposits between soil and trees during approximately the first 4–5 years after the accident^14–19^. Post-Chernobyl studies and the International Atomic Energy Agency (IAEA) suggest that after this early phase, the dynamics of ^137^Cs in forests tend to reach a quasi-equilibrium state, characterized by a balanced flux of ^137^Cs between soil and trees^20–22^. The ^137^Cs cycle in forests affected by the FDNPP accident is also believed to be approaching a quasi-equilibrium state over time^3^, and recent observational studies have suggested that this state has already been reached in some forests in Fukushima Prefecture^14,17,23^.

More than a decade after the FDNPP accident, the effects of forest contamination by ^137^Cs persist, such as the continued suspension of bed-log shipments for shiitake mushroom (*Lentinula edodes*) cultivation^3^. To resume forestry activities in ^137^Cs-contaminated areas, it is critical to assess the transfer of ^137^Cs from soil to trees. To quantify the transfer of ^137^Cs from forest soil to various types of vegetation, the aggregated transfer factor (*T*_*ag*_ (m^2^/kg)) under quasi-equilibrium conditions is one of the practical indicators^22^, defined as the ^137^Cs activity concentration (Bq/kg) in aboveground tree tissues divided by the total ^137^Cs inventory (Bq/m^2^) in soil. However, it is not yet sufficiently understood if or when forests affected by the FDNPP accident reached this quasi-equilibrium state or after how many years following the accident the *T*_*ag*_ can be considered appropriate for assessing the long-term transfer of ^137^Cs from forest soil to trees.

In addition, comparing the *T*_*ag*_ values obtained after the FDNPP accident with data obtained after the Chernobyl accident is important for generalizing the findings from the FDNPP accident and applying them to regions that have not experienced nuclear accidents. A comparison between Fukushima and Chernobyl has already been performed in the IAEA Modelling and Data for Radiological Impact Assessments (MODARIA II) Programme^24^. However, in that comparison, the Chernobyl dataset included *T*_*ag*_ values measured 5–15 years after the accident^25^, corresponding to a period assumed to represent quasi-equilibrium conditions. In contrast, Fukushima dataset provisionally employed values from 2015, which was only 4 years after the FDNPP accident^26^. However, it has not been assessed whether forests in Fukushima had reached a quasi-equilibrium state by that time. In this study, the MODARIA II comparison is used as an example to illustrate the limitations of such cross-accident comparisons, particularly regarding the assumption of quasi-equilibrium conditions. Therefore, it is necessary to verify the validity of the *T*_*ag*_ comparison in the previous study^24^.

To evaluate the quasi-equilibrium state of a forest, one approach is to examine the stability of the total ^137^Cs inventory in the aboveground compartments (needles/leaves, branches, stem bark (hereafter, bark), and stem wood) after correcting for the physical decay of ^137^Cs. If the total ^137^Cs inventory in these compartments remains stable over time, this suggests that the flux of ^137^Cs from trees to soil and that from soil to trees are approximately balanced. However, a previous study has indicated that the ^137^Cs distribution changed considerably from 2011 to 2015^27^, and no sufficiently long dataset has been reported to evaluate the stability of the total ^137^Cs inventory in the aboveground compartments. Although another study has reported the total ^137^Cs inventory in the aboveground compartments from 2014 to 2020, a key limitation is the lack of data from the early post-accident period (2011–2013)^28^. Furthermore, it is also important to evaluate whether the distribution of ^137^Cs in the aboveground compartments has remained stable. Unless the proportional distribution of ^137^Cs in the aboveground compartments—defined as the proportion of ^137^Cs inventory in each compartment (needles/leaves, branches, bark, sapwood, and heartwood) relative to the total aboveground ^137^Cs inventory—remains stable, it cannot be concluded that *T*_*ag*_ after the FDNPP accident has reached a level comparable to that observed after the Chernobyl accident. Therefore, assessments based on long-term observations, including those from the early post-accident period, are necessary.

The only research group in Japan that has monitored ^137^Cs distribution in forests for over 10 years since the FDNPP accident is the Forestry and Forest Products Research Institute (FFPRI)^14,17^, together with the Forestry Agency of Japan. Previous studies^27,29^ have reported the total ^137^Cs inventory in the aboveground compartments from 2011 to 2015. In addition, ^137^Cs activity concentrations in bark and stem wood from 2011 to 2020^17^ and in needles/leaves and branches from 2016 to 2019^30^ have been published.

The objective of this study was to assess when the ^137^Cs cycle in the forests first reached a quasi-equilibrium state after the FDNPP accident, based on the analysis of 10 years of data on the total ^137^Cs inventory in the aboveground compartments and its proportional distribution. In addition, we evaluated the validity of the *T*_*ag*_ comparison between Chernobyl and Fukushima. These analyses are expected to enhance the understanding of the long-term dynamics of ^137^Cs in forests and promote further efforts toward the resumption of forestry. In this study, we newly obtained aboveground biomass data from 2016 to 2020 as well as ^137^Cs activity concentrations in needles/leaves and branches for 2020, with additional concentration data obtained between 2017 and 2019. The total ^137^Cs inventory in the aboveground compartments and its ^137^Cs proportional distribution after 2015, calculated from both newly obtained and previously reported data, allowed us to determine the time at which the ^137^Cs cycle in the forests first reached a quasi-equilibrium state and to evaluate the stability of the ^137^Cs distribution in the aboveground compartments.

## Materials and methods

### Information on monitoring sites

**Fig. 1:**
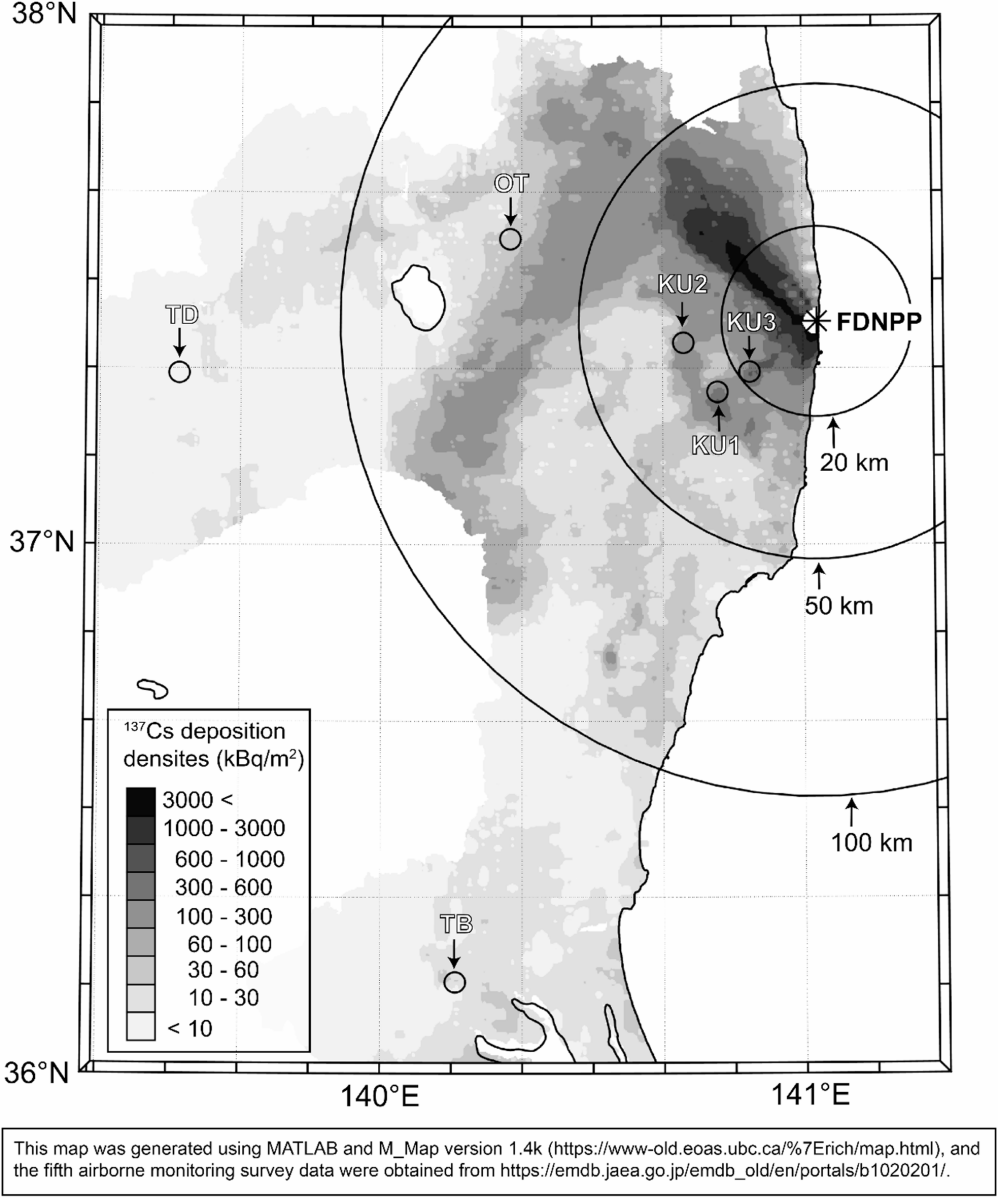
Map displaying the locations of the monitoring sites in this study. Black open circles indicate monitoring sites, while gray shading represents the distribution of ^137^Cs deposition in Fukushima and Ibaraki Prefectures on June 28, 2012 (fifth airborne monitoring survey).

This study was conducted at five sites in Fukushima Prefecture (sites KU1–3 in Kawauchi Village, site OT in Otama Village, and site TD in Tadami Town) and one site in Ibaraki Prefecture (site TB in Ishioka City), Japan (Fig. 1), consistent with previous studies^14,17^. Sites KU1 and OT each contained three monitoring plots, yielding a total of 10 plots in this study. The dominant tree species in each plot were Japanese cedar (*Cryptomeria japonica*; KU1-S, KU2-S, OT-S, and TD-S), Japanese cypress (*Chamaecyparis obtusa*; KU1-H and TB-H), konara oak (*Quercus serrata*; KU1-Q and OT-Q), and Japanese red pine (*Pinus densiflora*; KU3-P and OT-P). Because plot KU3-P contained not only Japanese red pine but also deciduous broad-leaved trees, Japanese chestnut (*Castanea crenata*) was also included in the ^137^Cs distribution survey. The plot notation followed that established by Manaka et al.^14^, and detailed information (latitude, longitude, elevation, stand age, and plot area) is provided in Table 1.

**Table 1:** General information for 10 monitoring plots in Fukushima and Ibaraki Prefectures.

	Kawauchi Village					Otama Village			Tadami Town	Mt. Tsukuba
Plot name	KU1-S	KU1-H	KU1-Q	KU2-S	KU3-P	OT-S	OT-Q	OT-P	TD-S	TB-H
Latitude	37°17’N	37°17’N	37°17’N	37°23’N	37°20’N	37°35’N	37°34’N	37°34’N	37°19’N	36°10’N
Longitude	140°48’E	140°48’E	140°48’E	140°43’E	140°52’E	140°18’E	140°18’E	140°19’E	139°31’E	140°11’E
Elevation (m)	660	720	720	690	610	730	760	750	790	360
Dominant species	*Cryptomeria japonica*	*Chamaecyparis obtusa*	*Quercus serrata*	*Cryptomeria japonica*	*Pinus densiflora*	*Cryptomeria japonica*	*Quercus serrata*	*Pinus densiflora*	*Cryptomeria japonica*	*Chamaecyparis obtusa*
Stand age in 2011 (year)	43	26	26	55	45	42	43	43	38	43
Plot area (ha)	0.16	0.10	0.06	0.12	0.24	0.24	0.24	0.24	0.21	0.06

### Assessment of aboveground biomass

The plot-scale biomass (kg/m^2^) of each aboveground compartment (needles/leaves, branches, bark, sapwood and heartwood) from 2016 to 2020 was obtained using the method employed in previous studies^27,29^. To calculate the biomass of needles/leaves and branches, we measured the diameter at breast height (DBH) for all mature trees (DBH > approximately 10 cm) within each monitoring plot (Supplementary Table S1). Trees that died during the observation period were excluded from the measurements, and trees whose DBH exceeded approximately 10 cm during the observation period were newly included. The biomass of needles/leaves and branches for each tree (kg) was calculated using previously reported allometric equations^32^ based on DBH data. The biomass of bark, sapwood, and heartwood was obtained by multiplying the total stem volume by the respective volume ratios and specific densities (Supplementary Table S2). This total stem volume was calculated using an equation^33^ with DBH and tree height as explanatory variables. Tree height was estimated from DBH using an existing equation^32^, except for Japanese cypress at site TB, where tree height measurements were conducted^34^. The plot-scale aboveground biomass (kg/m^2^) of the main tree species (Japanese cedar, Japanese cypress, Japanese red pine, konara oak, and Japanese chestnut) was determined by dividing the total biomass of each species within the monitoring plot (kg) by the plot area (m^2^) (Supplementary Table S3).

### Sampling and measurement of ^137^Cs activity concentration

Although the ^137^Cs activity concentrations in needles/leaves and branches from 2016 to 2019 have been reported by Kenzo et al.^30^, additional data for this period are newly reported in this study, including those for konara oak at site KU1 (2017–2019), Japanese cedar at sites KU1 and KU2 (2017), Japanese cypress at site KU1 (2017), and Japanese chestnut at site KU3 (2017–2019). All ^137^Cs activity concentrations in needles/leaves and branches for 2020 were obtained in this study. Three trees of different diameter classes (i.e., small, medium, and large), with diameter ranges reported in Ohashi et al. (2022)^17^, were felled near each monitoring plot, and needles/leaves and branches were obtained from the crown, following the method described by Imamura et al.^27^. Since 2017, in several monitoring plots (Japanese cedar at sites KU1 and KU2, Japanese cypress at site KU1, and konara oak at site KU1), needles/leaves and branches were sampled by climbing trees or using a long sickle that reached the tree crown. Some of the collected samples (needles and branches) were divided into current-year and older (> 1 year) parts. Details are provided in Supplementary Table S4 and described in Kenzo et al.^30^. The collection and use of tree samples were conducted in accordance with all relevant guidelines, and all sampling was performed with permission from the Kanto Regional Forest Office of the Forestry Agency and the Kawauchi Village Office.

Before measuring the ^137^Cs activity concentrations, all homogenized needles/leaves and branches were oven-dried at 75 °C for 72 h. The samples were then packed into either a 100-mL (U8) container or a 0.7-L or 2-L Marinelli container. The ^137^Cs activity concentrations of needles/leaves and branches were measured at FFPRI using Ge detectors (GEM20-70, GEM40P4-76, GEM-FX7025P4-ST, and GWL-120-15-LB-AWT, ORTEC, Oak Ridge, USA). The peak efficiencies of the Marinelli and U8 containers were calibrated using standard sources (MX033MR and MX033U8PP; Japan Radioisotope Association, Tokyo, Japan). The measurement times ranged from 1,800 s (30 m) to 85,800 s (approximately 1 d), and the maximum counting error was less than 10%. All ^137^Cs activity concentrations in this study were decay-corrected to September 1, 2020.

### Data and ^137^Cs inventory in aboveground compartments

In this study, we employed plot-scale biomass data for each aboveground compartment (needles/leaves, branches, bark, sapwood and heartwood) from 2011 to 2015, as reported in previous studies^27,29^. These studies also reported ^137^Cs activity concentrations in needles/leaves and branches from 2011 to 2015^27,29^ and in bark, sapwood, and heartwood from 2011 to 2020^17,31^. In addition, Kenzo et al.^30^ reported ^137^Cs activity concentrations in needles/leaves and branches from 2016 to 2019. The arithmetic mean of the ^137^Cs activity concentration in each aboveground compartment (needles/leaves, branches, bark, sapwood, and heartwood) was calculated using both previously reported data and the ^137^Cs activity concentrations in needles/leaves and branches obtained in this study (Supplementary Table S5). In this study, the ^137^Cs activity concentration in the entire needle compartment in evergreen coniferous species was calculated as a weighted average, accounting for needle longevity. For Japanese cedar and Japanese cypress, current-year and older needles were weighted 20% and 80%, respectively, corresponding to reported needle longevities of 4–6 years^35,36^. In contrast, for Japanese red pine, weights of 33% and 67% were applied, corresponding to a needle longevity of approximately 2–4 years^37^. We employed the ^137^Cs activity concentration in older branches to represent the concentration of entire branch compartment, as the contribution of current-year branches was assumed to be small. The ^137^Cs inventory (Bq/m^2^) in the aboveground compartments of the main tree species (Japanese cedar, Japanese cypress, Japanese red pine, konara oak, and Japanese chestnut) was calculated by multiplying the plot-scale biomass (kg/m^2^) by the arithmetic mean of ^137^Cs activity concentration (Bq/kg) (Supplementary Table S5), following the approach of previous studies^27,29^.

### Calculation of *T*_*ag*_

*T*_*ag*_ for needles/leaves and stem wood was calculated by dividing the geometric mean of ^137^Cs activity concentrations in these compartments by the geometric mean of ^137^Cs inventory in forest soil, which included both the organic horizon and the 0–20-cm mineral soil horizon (Supplementary Table S6). We employed ^137^Cs inventory data for forest soil (decay-corrected to September 1, 2020) reported by Manaka et al.^14^. In that study^14^, the mineral soil horizon was investigated to a depth of 0–20 cm at only three sites during 2011–2013, and at four sites during 2014–2020, whereas at the remaining sites, measurements were limited to the upper 0–5 cm. Therefore, for sites where measurements were limited to 0–5 cm, the data were adjusted using the geometric mean of the ratio—calculated separately for each site and for each survey year—between the total ^137^Cs inventory in the organic horizon plus the 0–5-cm mineral soil horizon and that in the organic horizon plus the 0–20-cm mineral soil horizon, based on data from sites with full-depth measurements^14^. After applying this correction, the geometric mean was calculated using the ^137^Cs inventory data for all sites and used as the denominator for calculating *T*_*ag*_. The *T*_*ag*_ values for needles/leaves and stem wood are presented in Supplementary Table S7.

The data summarized in the Supplementary Information represent the latest data obtained by the FFPRI group over the 10 years following the FDNPP accident, including previously reported data^14,17,27,29–31^.

### Temporal trend analysis

Time series trends were evaluated using a dynamic linear model (DLM)^38^. A basic DLM is expressed by the following three equations:

1 $$Y_{t} = \mu_{t} + \epsilon_{t} ,\epsilon_{t} \sim {\text{ }}Normal{\text{ }}(0,\sigma_{\epsilon} ^{2} )$$

2 $$\mu_{t} = \mu_{{t - 1}} + \beta_{{t - 1}} + \eta_{t} ,\eta_{t} \sim Normal{\text{ }}(0,\sigma_{\eta} ^{2} )$$

3 $$\beta_{t} = \beta_{{t - 1}} + \xi_{t} ,\xi_{t} \sim Normal{\text{ }}(0,\sigma_{\xi} ^{2} )$$

where *Y*_*t*_ represents the observed values at time *t*; *µ*_*t*_ and *β*_*t*_ denote the level (true state) and trend (slope), respectively; and *ε*_*t*_, *η*_*t*_, and *ξ*_*t*_ represent the corresponding noise terms. To capture longer-term trends, we applied the smooth trend model^17^, in which both *µₜ* and *βₜ* are influenced by a common noise component. By integrating Eqs. (2) and (3), the expression for *µₜ* in the smooth trend model can be derived as follows:

4 $$\mu _{t} = {\text{ }}2\mu _{{t - 1}} - \mu _{{t - 2}} + \eta _{t} ,\eta _{t} \sim Normal{\text{ }}(0,\sigma _{\eta } ^{2} )$$

Bayesian estimation employing a Markov chain Monte Carlo (MCMC) approach was employed to determine the parameters (*µ*_*t*_, *σ*_*ε*_^*2*^, and *σ*_*η*_^*2*^). The cmdstanr package (version 0.8.0) in R (version 4.5.1)^39^ was utilized for Bayesian estimation. *Y*_*t*_ consisted of the log-transformed total ^137^Cs inventory (decay-corrected to September 1, 2020), the proportional distribution of ^137^Cs inventory in the aboveground compartments, and *T*_*ag*_. Four MCMC chains of 60,000 iterations were run, with the first 30,000 iterations treated as warm-up, yielding 120,000 samples per parameter. MCMC convergence was determined by verifying that the maximum *Rhat* value did not exceed 1.01 and that the number of divergent transitions after warm-up remained below 1200 (less than 1% of the total samples). If these conditions were not met, the number of iterations was increased to 70,000 (with the first 35,000 iterations used as warm-up), 100,000 (with the first 50,000 iterations used as warm-up), or 120,000 (with the first 60,000 iterations used as warm-up). DLM analysis was not performed for Japanese red pine and Japanese chestnut at site KU3, as they were surveyed for less than 5 years and the dataset did not include the early post-FDNPP accident period. To assess the stability of temporal variations, we evaluated whether the 95% credible interval for the difference between the 2020 level (*µ*_2020_) and those of other years included zero.

### Comparison with post-Chernobyl *T*_*ag*_ data

Post-Chernobyl *T*_*ag*_ values for needles/leaves and stem wood were obtained from the same dataset used in the MODARIA II Programme^24^ and reported in IAEA-TECDOC-1616^25^. These data were collected in various European countries, including Austria, Belarus, Belgium, Croatia, Denmark, Ireland, Italy, Russia, Sweden, Switzerland, and the UK, between 1991 and 2001. For the data after the FDNPP accident, we employed *T*_*ag*_ values from the period during which the total ^137^Cs inventory in the aboveground compartments, the proportional distribution of ^137^Cs in the aboveground compartments, and the time-dependent changes in *T*_*ag*_ were confirmed to be stable in this study. The geometric mean of the *T*_*ag*_ values for needles/leaves and stem wood for each year during that period was treated as a single data point (i.e., each monitoring plot had only one *T*_*ag*_ value for needles/leaves and stem wood). We also included the 2015 post-FDNPP *T*_*ag*_ data^26^ used in the MODARIA II Programme^24^ as reference values for comparison. Differences in *T*_*ag*_ for needles and stem wood in coniferous species between Chernobyl and Fukushima were assessed using boxplots displaying the 95% confidence interval of the median. These boxplots were created using the boxchart function in MATLAB.

## Results

### Temporal changes in total ^137^Cs inventory in the aboveground compartments

**Fig. 2:**
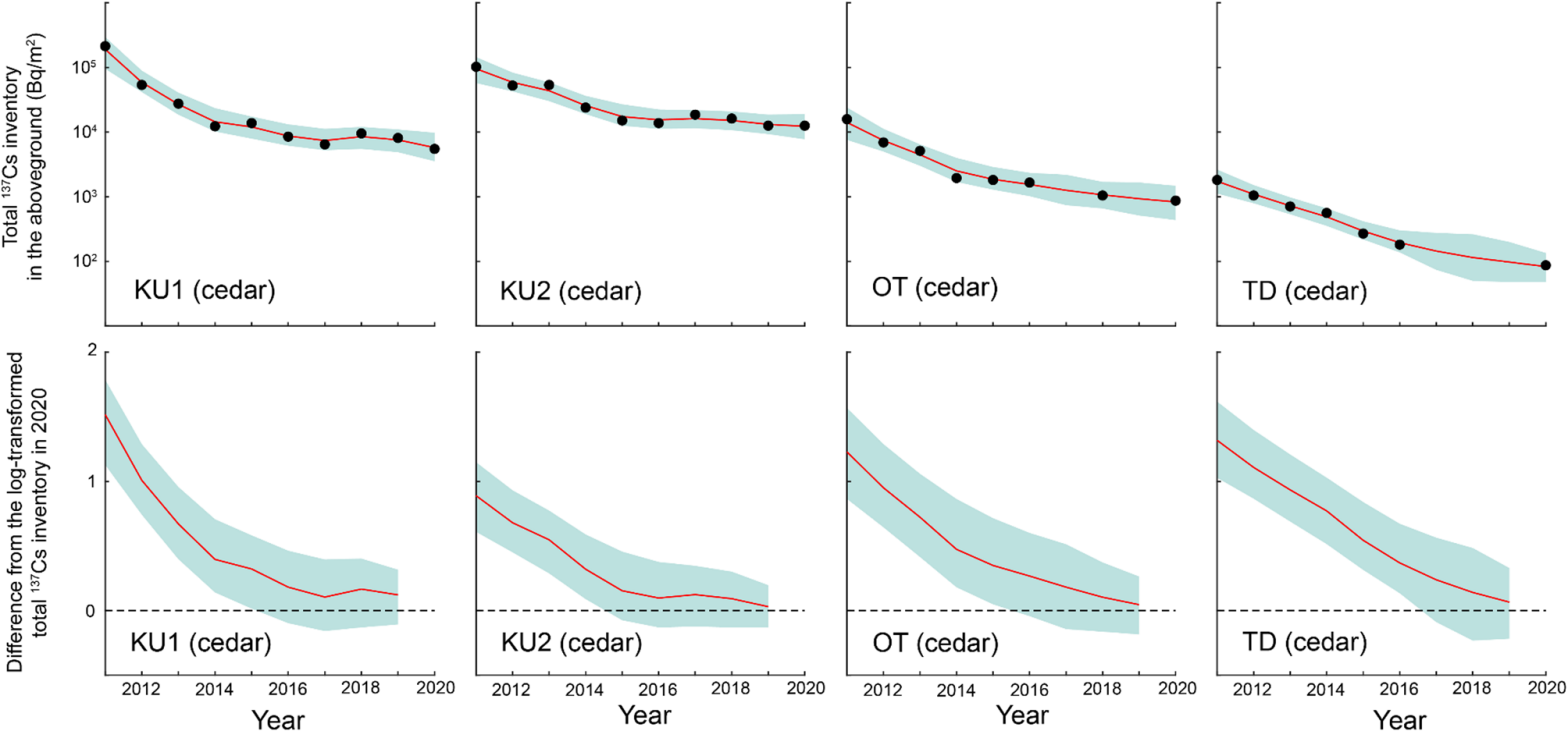
Temporal variations in the total ^137^Cs inventory (decay-corrected to September 1, 2020) in the aboveground compartments (needles, branches, bark, and stem wood) of Japanese cedar at four study sites: KU1, KU2, OT, and TD. Black circles indicate the observed values, while red lines indicate the dynamic linear model fit. Blue shaded areas represent 95% credible intervals. The lower four panels display the differences from the log-transformed total ^137^Cs inventory in 2020 (where a value of 1 corresponds to a 10-fold increase) for each model state, based on data from 2011 to 2019. Each panel includes the difference (red line) and the 95% credible interval (blue shading).

**Fig. 3:**
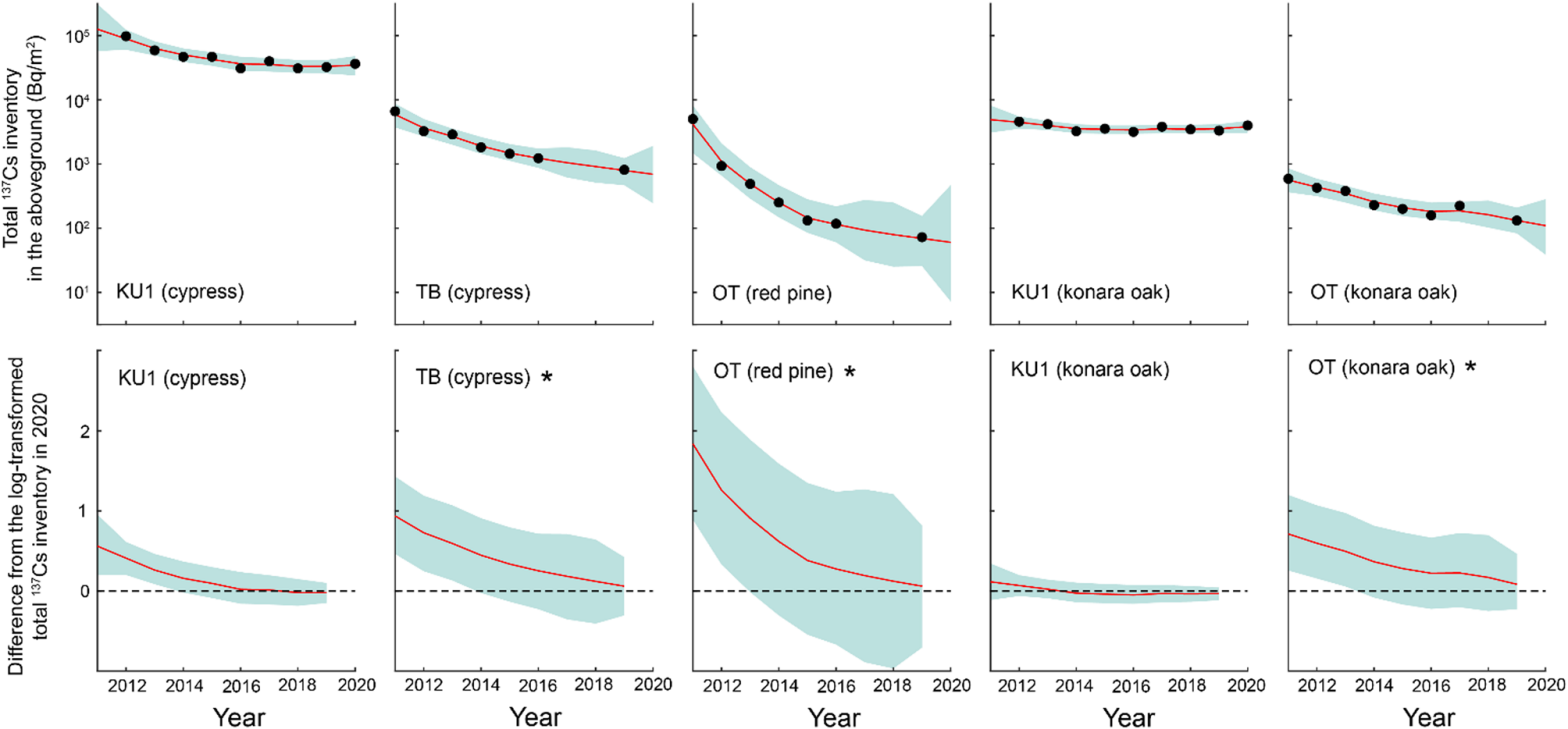
Temporal variations in the total ^137^Cs inventory (decay-corrected to September 1, 2020) in the aboveground compartments (needles/leaves, branches, bark, and stem wood) of Japanese cypress, Japanese red pine, and konara oak at sites KU1 (Japanese cypress), TB (Japanese cypress), OT (Japanese red pine), KU1 (konara oak), and OT (konara oak). Black circles represent observed values, while the red lines indicate the dynamic linear model fit. Blue shaded areas represent 95% credible intervals. The lower five panels display deviations from the log-transformed total ^137^Cs inventory in 2020 for each model state, based on data from 2011 to 2019. Each panel includes the difference (red line) and the 95% credible interval (blue shading). *Note*: The Japanese cypress data at site TB for 2011 and 2012 were actually obtained in February 2012 and January 2013, respectively. For sites marked with an asterisk (*) in the lower panels, the 2020 baseline values are extrapolated estimates. Consequently, the 95% credible intervals for the differences tend to be larger, and differences may lose statistical significance from zero earlier. Therefore, caution is advised when interpreting these results.

The total ^137^Cs inventory (decay-corrected to September 1, 2020) in the aboveground compartments of Japanese cedar at site KU1, observed annually from 2011 to 2020, exhibited no statistically significant difference from the 2020 level during the period from 2016 to 2019 (Fig. 2); here, a difference was considered non-significant when the 95% credible interval for the difference between the 2020 level and those of other years included zero. For site KU2, no statistically significant difference was observed from 2015 to 2019. Site OT exhibited a trend similar to that observed at site KU1, whereas at site TD, no significant difference from the 2020 level was observed during 2017–2019. Therefore, the period during which the total ^137^Cs inventory in the aboveground compartments remained stable was 2017–2020 across all four Japanese cedar sites. For Japanese cypress and Japanese red pine, which are also evergreen coniferous species, a similar trend was observed, with the difference from the 2020 level decreasing over time (Fig. 3). At site KU1, the total ^137^Cs inventory in the aboveground compartments of konara oak exhibited no statistically significant difference from the 2020 level during 2011–2019 (Fig. 3). In contrast, at site OT, where observations have been conducted since 2011, significant differences from the 2020 level were observed during 2011–2013.

Assessment of all tree species suggested that although there were slight differences among monitoring sites, the log-transformed total ^137^Cs inventory in the aboveground compartments remained stable from 2017 to 2020.

### Proportional distribution of ^137^Cs inventory in the aboveground compartment

**Fig. 4:**
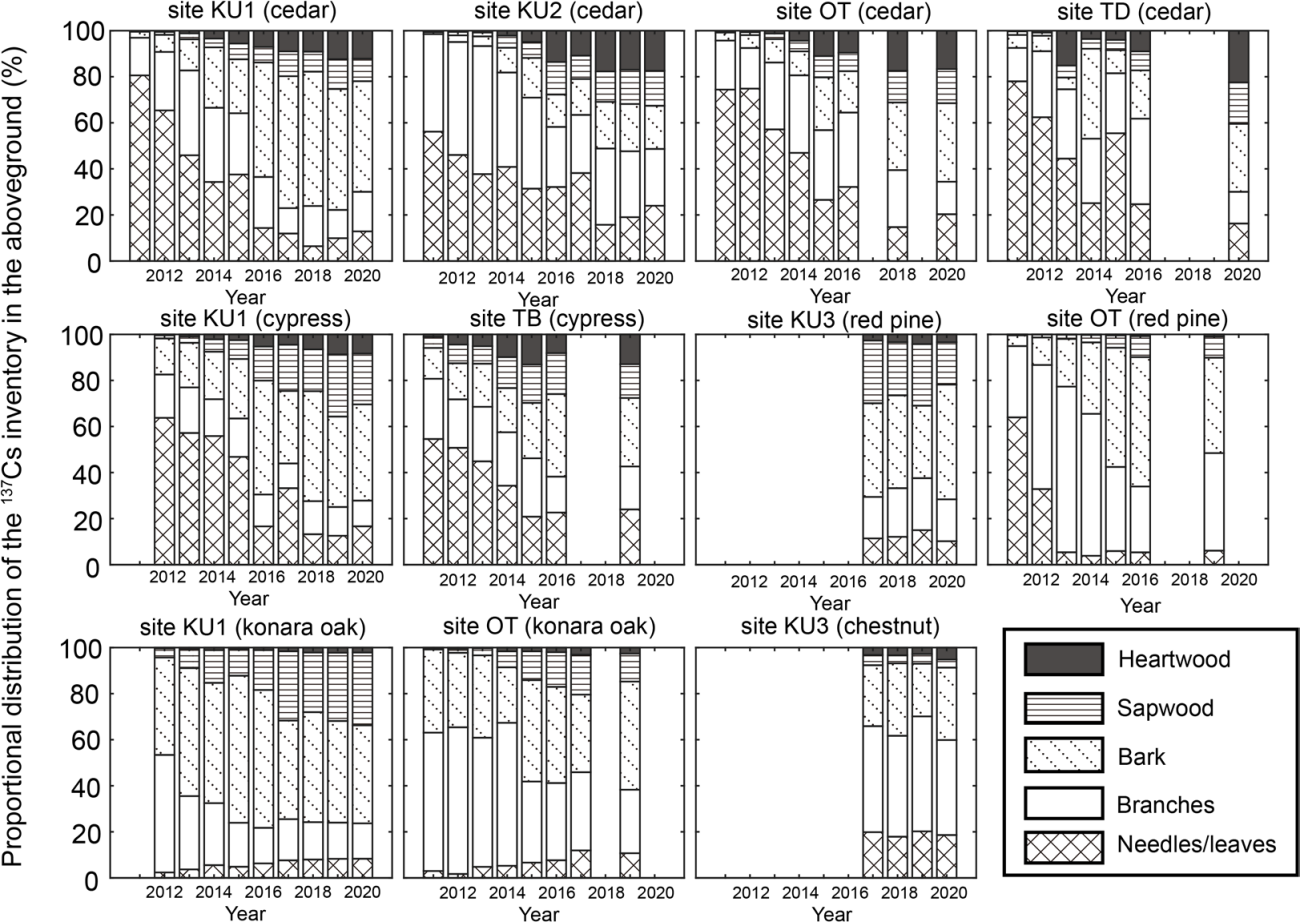
Temporal variations in the proportional distribution of ^137^Cs inventory in the aboveground compartments from 2011 to 2020.

In 2011 and 2012, needles accounted for the largest proportion of the proportional distribution of ^137^Cs inventory in evergreen coniferous trees (i.e., Japanese cedar, Japanese cypress, and Japanese red pine); however, this proportion decreased over time (Fig. 4). For deciduous broad-leaved trees, such as konara oak and Japanese chestnut, branches and bark accounted for a large proportion in 2011 and 2012, and this proportion also decreased over time. In contrast, a gradual increase in the proportion of leaves and stem wood was observed over time.

**Fig. 5:**
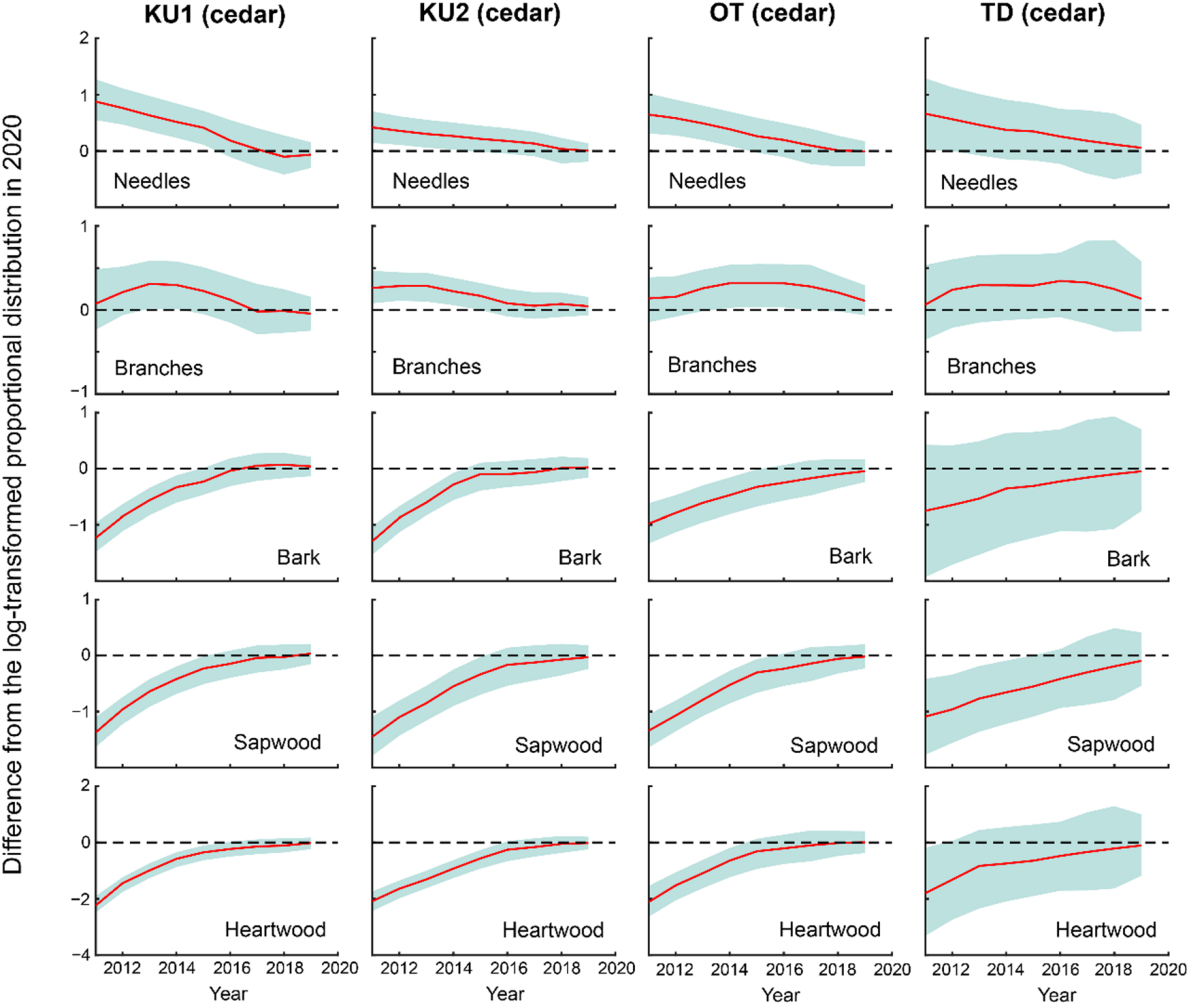
Differences from the log-transformed proportional distribution of ^137^Cs inventory in the aboveground compartments in 2020 for each model state at four Japanese cedar sites (KU1, KU2, OT, and TD), based on data from 2011 to 2019. The red lines indicate differences, while the blue shaded areas represent 95% credible intervals.

**Fig. 6:**
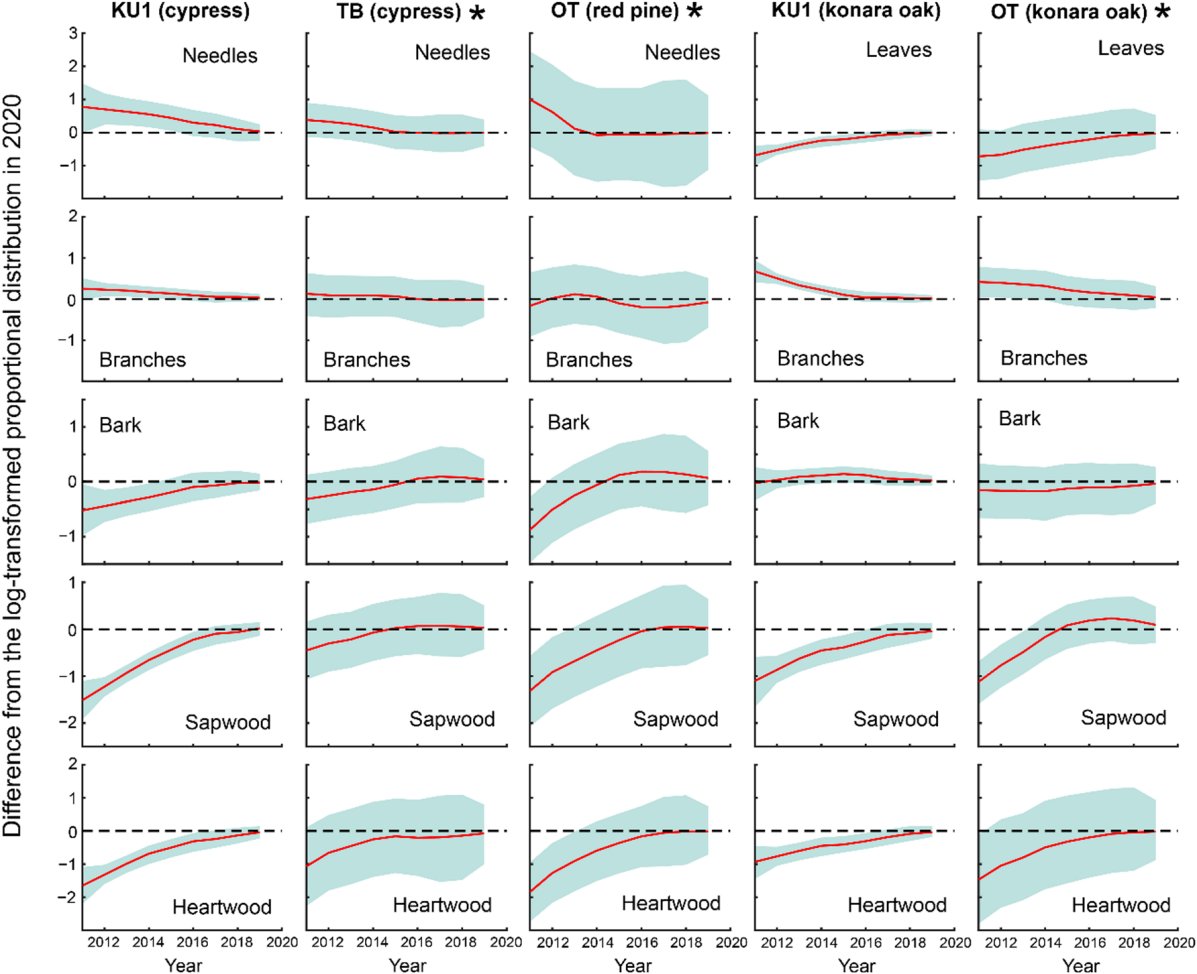
Differences from the log-transformed proportional distribution of ^137^Cs inventory in the aboveground compartments in 2020 for each model state at Japanese cypress, Japanese red pine, and konara oak sites, based on data from 2011 to 2019. Red lines represent differences, while blue shaded areas represent 95% credible intervals. *Note*: For sites marked with an asterisk (*), the 2020 baseline values are extrapolated estimates. Consequently, the 95% credible intervals for the differences tend to be larger, and the differences may lose statistical significance from zero earlier. Therefore, caution is advised when interpreting these results.

Figures 5 and 6 display the deviations in the log-transformed proportional distribution of ^137^Cs inventory in the aboveground compartments relative to 2020 for each model state. The DLM fitting results are provided in Supplementary Figs. S1 and S2. At the four Japanese cedar sites, the proportion of needles decreased over time, and by 2016, no significant differences from the 2020 level were observed at any of the sites (Fig. 5). Similarly, for branches, no significant differences from the 2020 level were observed at any of the sites after 2017. In contrast, bark, sapwood, and heartwood exhibited increasing trends over time, stabilizing 2016, after which their proportions did not differ significantly from the 2020 level at any of the sites. Likewise, for Japanese cypress, Japanese red pine, and konara oak, no significant differences from the 2020 level were observed at any of the sites after 2017 across all above-ground compartments, including needles/leaves, branches, bark, sapwood, and heartwood (Fig. 6). These findings indicate that the aboveground proportional distribution of ^137^Cs has remained stable since at least 2017, although the timing of this stabilization varied among monitoring sites and tree species.

### *T*_*ag*_ for needles/leaves and stem wood following the FDNPP accident

**Fig. 7:**
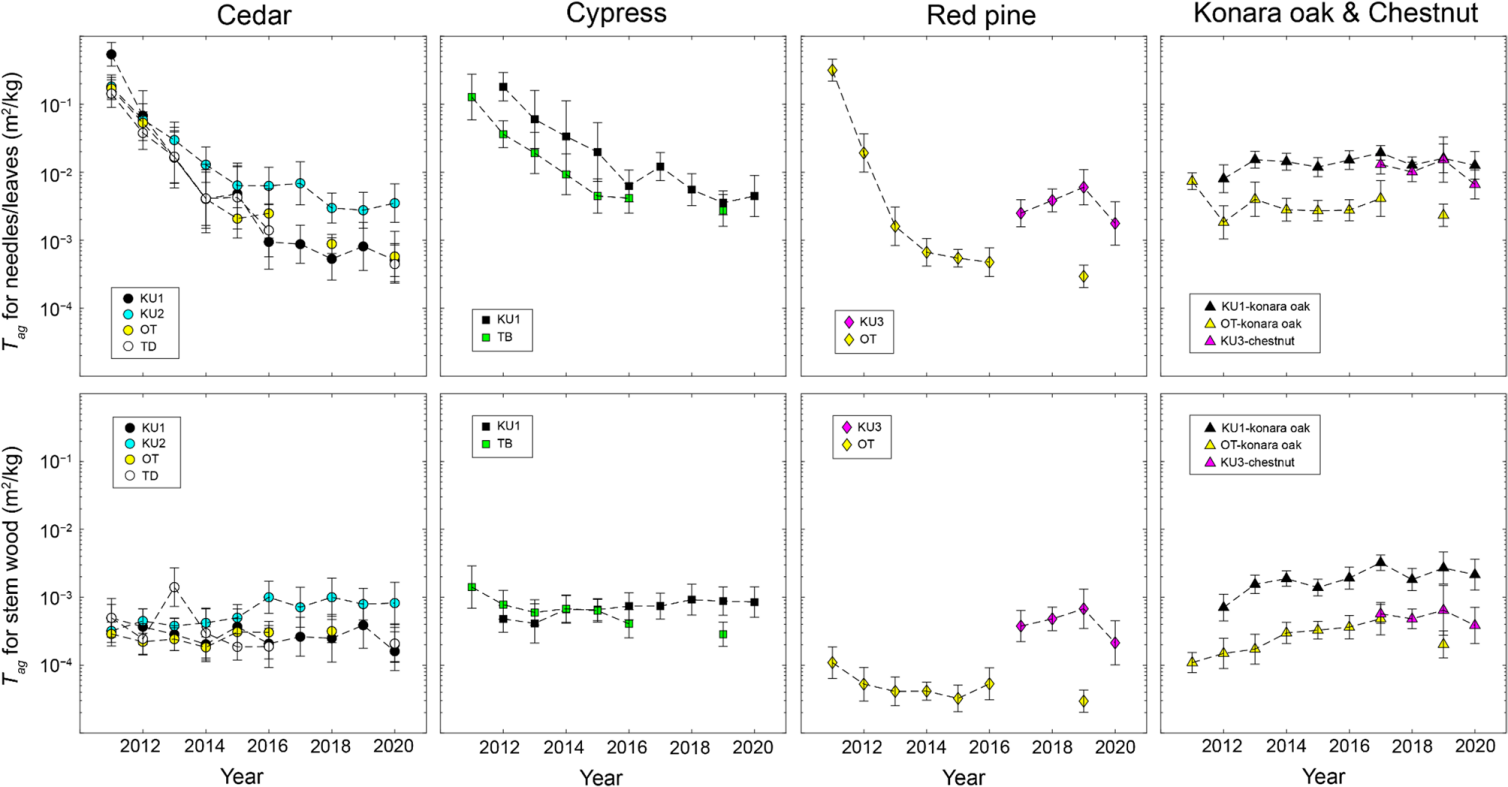
Temporal variations in the geometric mean of *T*_*ag*_ for needles/leaves and stem wood. Error bars represent the geometric mean multiplied and divided by the geometric standard deviation.

**Fig. 8:**
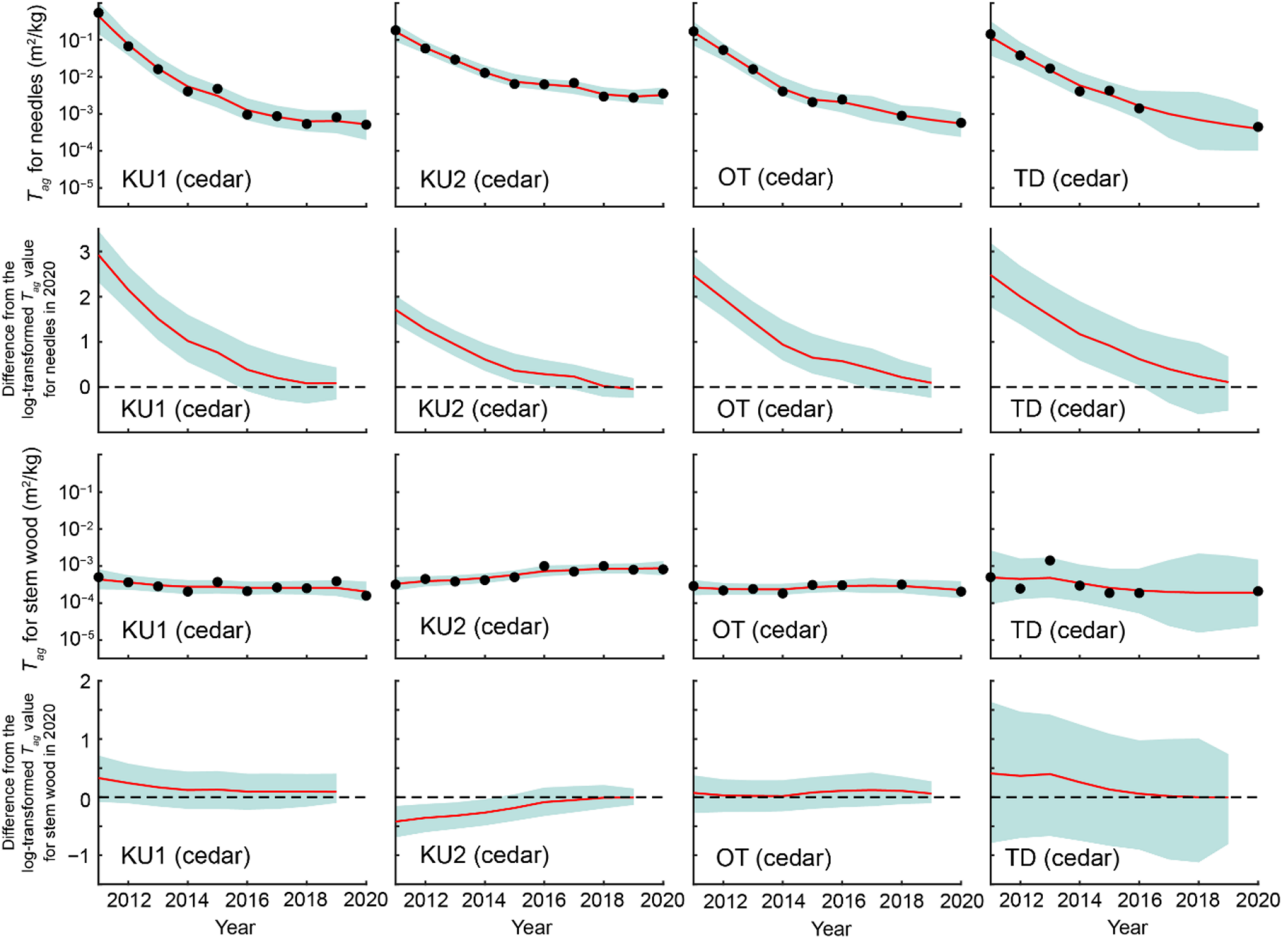
Results of fitting the dynamic linear model (DLM) to the geometric mean of *T*_*ag*_ for needles and stem wood of Japanese cedar at four study sites. Black circles represent the observed values, while red lines represent the DLM fit. Blue shaded areas indicate 95% credible intervals. The panels below each DLM fitting result display the differences from the log-transformed *T*_*ag*_ in 2020 for each model state, based on data from 2011 to 2019. Each panel includes the difference (red line) and the 95% credible interval (blue shaded area).

**Fig. 9:**
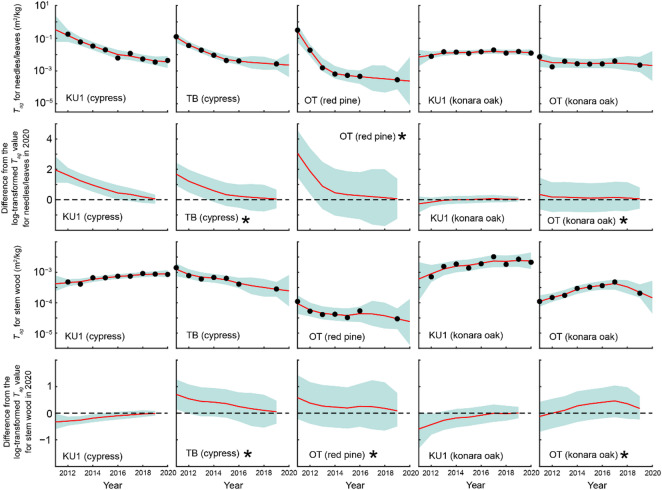
Results of fitting the dynamic linear model (DLM) to the geometric mean of *T*_*ag*_ for needles/leaves and stem wood at Japanese cypress, Japanese red pine, and konara oak sites. Black circles represent the observed values, while red lines represent the DLM fit. Blue shaded areas denote 95% credible intervals. The panels below each DLM fitting result present the differences from the log-transformed *T*_*ag*_ in 2020 for each model state, based on data from 2011 to 2019. Each panel includes the difference (red line) and 95% credible interval (blue shaded area). *Note*: For sites marked with an asterisk (*), the 2020 baseline values are extrapolated estimates. Consequently, the 95% credible intervals for the differences tend to be larger, and the differences may lose statistical significance from zero earlier. Therefore, caution is advised when interpreting these results.

Temporal variations in *T*_*ag*_ for needles/leaves and stem wood are presented in Fig. 7. Fitting the data using a DLM revealed that the *T*_*ag*_ values for needles at all four Japanese cedar sites exhibited no significant differences from the 2020 levels from 2017 onward (Fig. 8). Similarly, the *T*_*ag*_ values for stem wood did not differ significantly from the 2020 levels after 2015. For both needles/leaves and stem wood of other tree species, the *T*_*ag*_ values likewise exhibited no statistically significant differences from the 2020 levels from 2017 to 2015 onward, respectively (Fig. 9). These results indicate that, since at least 2017, several aspects have remained stable. These include: (i) the total ^137^Cs inventory in the aboveground compartments, (ii) the ^137^Cs proportional distribution in the aboveground compartments, and (iii) the temporal variations in *T*_*ag*_ for needles/leaves and stem wood.

### Comparison of *T*_*ag*_ values between Chernobyl and Fukushima

**Fig. 10:**
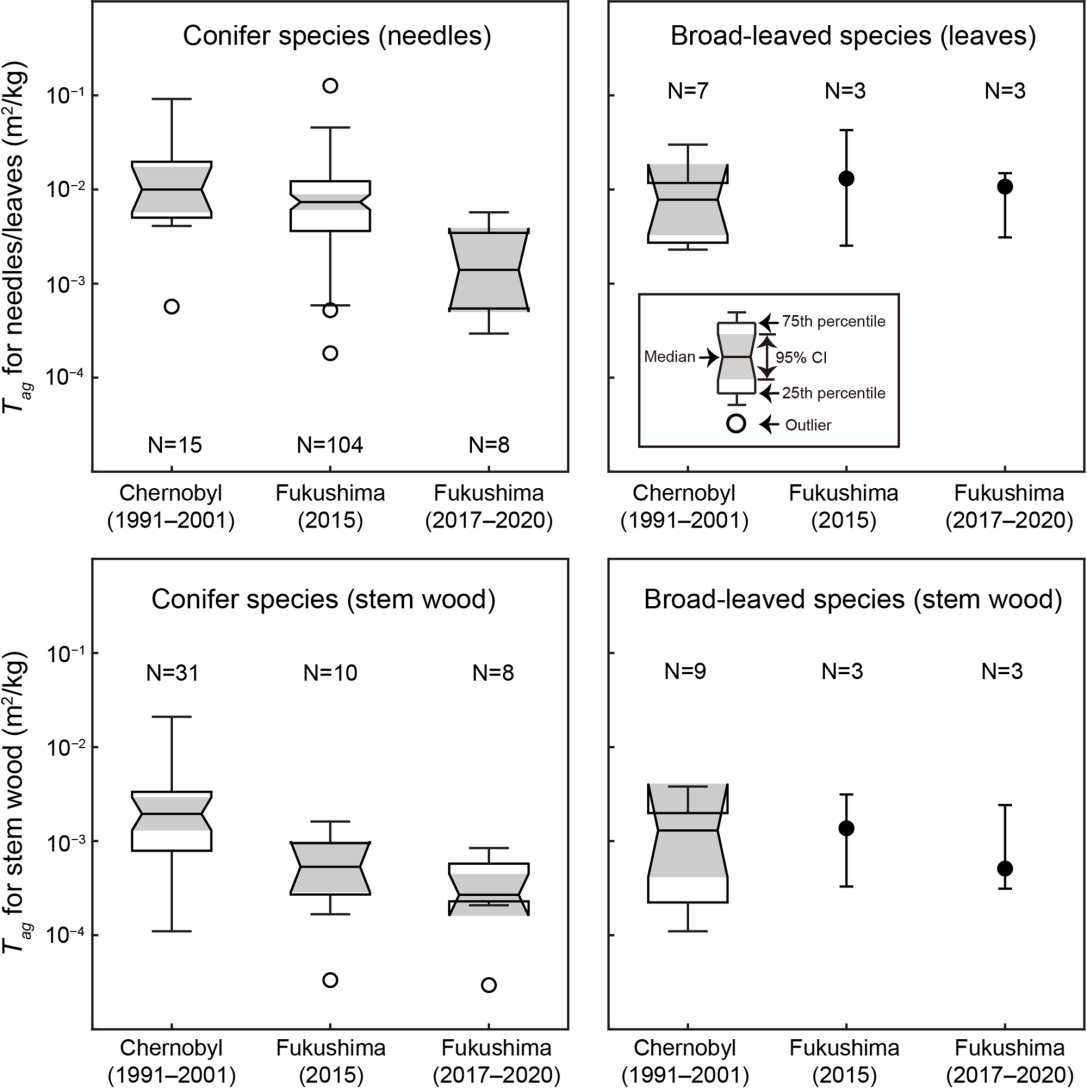
Comparison of needle/leaf and stem wood *T*_*ag*_ values between Chernobyl and Fukushima using boxplots. The gray shaded areas represent the 95% confidence intervals (CIs) of the medians, while the whiskers indicate data within 1.5 × the interquartile range. For broad-leaved species, because only three *T*_*ag*_ values were available for leaves and stem wood after the Fukushima nuclear accident, the median and range (minimum and maximum) are displayed instead. The 1991–2001 post-Chernobyl data^25^ and the 2015 post-Fukushima data^26^ were obtained from previous studies included in the MODARIA II Programme^24^.

Comparisons with Chernobyl data were performed using *T*_*ag*_ values obtained in this study for the period from 2017 to 2020 (Fig. 10). For evergreen conifers, the median *T*_*ag*_ for needles in Chernobyl (median = 1.00 × 10^− 2^; 95% confidence interval: 5.74 × 10^− 3^–1.74 × 10^− 2^) was significantly higher than that in Fukushima from 2017 to 2020 (median = 1.40 × 10^− 3^; 95% confidence interval: 5.01 × 10^− 4^–3.90 × 10^− 3^). The median *T*_*ag*_ for needles in Fukushima in 2015 (median = 7.36 × 10^− 3^; 95% confidence interval: 6.10 × 10^− 3^–8.87 × 10^− 3^) was not significantly different from that in Chernobyl but was significantly higher than the median *T*_*ag*_ observed in Fukushima from 2017 to 2020. In addition, we found that the median *T*_*ag*_ for stem wood was higher in Chernobyl (median = 1.95 × 10^− 3^; 95% confidence interval: 1.30 × 10^− 3^–2.92 × 10^− 3^) than in Fukushima from 2017 to 2020 (median = 2.69 × 10^− 4^; 95% confidence interval: 1.61 × 10^− 4^–4.48 × 10^− 4^). In contrast, the median *T*_*ag*_ for stem wood in Fukushima in 2015 (median = 5.35 × 10^− 4^; 95% confidence interval: 2.86 × 10^− 4^–9.98 × 10^− 4^) was not significantly different from the median values observed in Fukushima from 2017 to 2020 but was significantly lower than that in Chernobyl. The *T*_*ag*_ values for coniferous species in Chernobyl were approximately seven times higher than those in Fukushima from 2017 to 2020. For deciduous broad-leaved trees, no clear differences could be established because data were collected from only three locations after the FDNPP accident in this study.

## Discussion

### Time at which ^137^Cs dynamics in forests stabilized after the FDNPP accident and associated *T*_*ag*_

For both evergreen coniferous and deciduous broad-leaved species, the log-transformed total ^137^Cs inventory in the aboveground compartments from 2017 to 2019 did not differ significantly from the 2020 level (Figs. 2 and 3). This indicates that the ^137^Cs flows between soil and trees became approximately balanced, suggesting that the forest ^137^Cs cycle reached a quasi-equilibrium state by at least 2017. In addition, the proportional distribution of ^137^Cs in the aboveground compartments remained stable from 2017 to 2020 (Figs. 5 and 6), and this stability was further confirmed by the temporal variations in *T*_*ag*_ for needles/leaves and stem wood (Figs. 8 and 9). Therefore, *T*_*ag*_ values derived from samples collected more than approximately 6 years after the accident are recommended for assessing the long-term transfer of ^137^Cs from soil to trees. Accordingly, for comparison with Chernobyl *T*_*ag*_ values, the appropriate reference period is more than approximately 6 years after the FDNPP accident. However, variations were observed among monitoring plots in the timing at which the total ^137^Cs inventory in the aboveground compartments and its internal proportional distribution began to stabilize. Therefore, although this study concludes that the forest ^137^Cs cycle stabilized more than approximately 6 years after the FDNPP accident, this timescale may not apply uniformly to all study sites, and caution should be exercised when generalizing this finding.

The total ^137^Cs inventory in forest soil—measured at the same monitoring plots as in this study and including both the organic horizon and the 0–20-cm mineral soil horizon—also exhibited a generally stable trend between 2017 and 2020^14^. Although the ^137^Cs inventory in the organic horizon decreased from 2011 to 2020, inventory in the surface mineral horizon, which contains the largest ^137^Cs inventory in the forest^27^, has generally remained stable over the 10 years following the FDNPP accident^14^. These findings indicate that since approximately 2017, the distribution of ^137^Cs in the forests has been generally stable in both the aboveground and belowground compartments.

The above assessment suggests that the *T*_*ag*_ values obtained in 2015 from forests affected by the FDNPP accident that were employed in the MODARIA II Programme^24^ are not suitable for comparison with *T*_*ag*_ values collected 5–15 years after the Chernobyl accident^25^. However, the *T*_*ag*_ data from 2015 and earlier remain valuable for understanding the behavior of ^137^Cs during the early post-accident phase. In 2015, 4 years after the FDNPP accident, needles that had been directly contaminated ^137^Cs may have remained in the canopies of Japanese cedar and Japanese cypress, given their longevity of approximately 4–6 years^35,36^. It is also possible that the wash-off of directly contaminated ^137^Cs on the surfaces of branches and bark was incomplete in 2015, leaving residual effects of direct contamination. These possible factors suggest that the dynamics and distribution of ^137^Cs in forests had not yet stabilized by 2015. As a result, comparing *T*_*ag*_ values obtained 4 years after the FDNPP accident with those from Chernobyl is likely premature. The observation that the median *T*_*ag*_ value in needles in 2015 (following the FDNPP accident) was significantly higher than that observed from 2017 to 2020 (Fig. 10) further supports this interpretation.

Since 2017, when the ^137^Cs dynamics in the forests might have been stabilized, the median *T*_*ag*_ values for needles and stem wood in evergreen conifers following the FDNPP accident have been significantly lower than those observed after the Chernobyl accident (Fig. 10). As noted in the MODARIA II Programme^24^, this is likely because the Chernobyl data included measurements from hydromorphic soils, whereas the *T*_*ag*_ data in this study were derived from automorphic soils^14^. Calmon et al.^16^ similarly reported that differences in soil type considerably affect the transfer of ^137^Cs from soil to trees. Moreover, differences in tree species between Japan and Europe may also have influenced the results. Therefore, in future studies, comparisons within a common genus (e.g., *Pinus*) will be necessary. For deciduous broad-leaved trees, we found no significant differences between the Chernobyl and Fukushima data. However, the observed *T*_*ag*_ values were generally similar. These observations are generally consistent with the results of a previous comparison using Fukushima data from 2015^24^, which also demonstrated no substantial differences in *T*_*ag*_ values. To evaluate whether significant differences in *T*_*ag*_ values exist, additional data—particularly from needles/leaves and stem wood collected after 2017 and from more locations affected by the FDNPP accident—are required to address the current data limitations.

We also assumed that the *T*_*ag*_ values obtained 5 to 15 years after the Chernobyl accident^25^ were collected under quasi-equilibrium conditions; however, this assumption may require further verification. If it is incorrect, the *T*_*ag*_ values for conifer needles in particular may have been overestimated for the period following the Chernobyl accident. Although available data are limited, it is valuable to determine when the total ^137^Cs inventory in the aboveground compartments and its proportional distribution stabilized after the Chernobyl accident, using an approach similar to the DLM-based method employed in this study.

In this study, we evaluated the dynamics and stability of the ^137^Cs proportional distribution in the aboveground compartments based on observational data collected from 2011 to 2020. However, caution is warranted in assuming that the stability of the ^137^Cs dynamics and proportional distribution in the aboveground compartments observed since 2017 will continue in the future. This is because, even if the ^137^Cs activity concentration in stem wood remains constant, the total ^137^Cs inventory in stem wood may increase due to continued stem growth. Furthermore, it has been suggested that at several study sites, root uptake of ^137^Cs from soil to trees may still increase 10 years after the FDNPP accident^17^. Therefore, further time series analysis incorporating data from 2020 onwards is required.

In addition, further verification using alternative approaches is important to more thoroughly determine whether the ^137^Cs cycle in forests following the FDNPP accident has reached a quasi-equilibrium state since at least 2017. This is because the 95% credible intervals for differences from the 2020 levels evaluated in this study were wide, and small variations may have been interpreted as stability. One possible approach is to measure stable cesium (^133^Cs) in the samples examined in this study and compare the results with the ^137^Cs activity concentrations^21,23,28^. If the concentration ratios of ^133^Cs and ^137^Cs are found to remain constant across tree compartments (needles/leaves, inner bark, and stem wood) from 2017 to 2020, but to differ between compartments before 2016, this would support the time series analysis results of this study.

### Impact of assessing the stability of ^137^Cs dynamics on model prediction

Previous studies^14,23^ reported that the cycle of ^137^Cs in forests reached a quasi-equilibrium state approximately 10 years after the FDNPP accident. However, this study is the first to identify when this quasi-equilibrium state was likely reached. This finding may be useful for validating model performance and improving the long-term prediction accuracy of ^137^Cs activity concentration in stem wood, as the timing of the ^137^Cs flux balance between soil and trees varies across different models^40^. In addition, the amount of ^137^Cs transferred from the canopy to the forest floor through litterfall, throughfall, and stemflow approximately 6 years after the FDNPP accident may serve as an indicator of ^137^Cs uptake by trees from soil, a process that is difficult to observe directly. Sakashita et al.^19^ reported that from 2020 to 2023, the ^137^Cs flux from the canopy to the forest floor in two Japanese cedar plots (KU1-S and KU2-S) and one deciduous broad-leaved plot (KU1-Q) ranged from 0.4% to 1.6% of the initial deposition. Given the equilibrium condition where ^137^Cs fluxes from the tree to soil and soil to the tree balances, these fluxes can be interpreted as the amount of ^137^Cs transferred from soil to trees between 2020 and 2023, and can also be used to validate model performance^40^.

## Concluding remarks

In this study, we assessed the 10-year variation in the total ^137^Cs inventory in the aboveground compartments (needles/leaves, branches, bark, sapwood, and heartwood) and its proportional distribution across 10 evergreen coniferous and deciduous broad-leaved forests affected by the FDNPP accident from 2011 to 2020. Our findings indicate that the decay-corrected total ^137^Cs inventory in the aboveground compartments, as of September 1, 2020, has remained stable since at least 2017, suggesting that the forest ^137^Cs cycle reached a quasi-equilibrium state. The proportional distribution of ^137^Cs in the aboveground compartments was also stable from 2017 to 2020, with no statistically significant temporal changes in the *T*_*ag*_ values for needles/leaves and stem wood. Based on these results, we concluded that the dynamics and distribution of ^137^Cs in forests have remained stable since approximately 6 years after the FDNPP accident, and that *T*_*ag*_ values obtained after 2017 are suitable for evaluating the long-term soil-to-tree transfer of ^137^Cs. Comparison of the *T*_*ag*_ values for the needles and stem wood of evergreen conifers obtained from 2017 to 2020 with those measured after the Chernobyl accident indicated that the *T*_*ag*_ values for Chernobyl were approximately seven times higher. This finding provides a valuable reference for applying insights from the Fukushima and Chernobyl accidents to regions that have not experienced nuclear accidents. Moreover, this study is the first to assess when the dynamics of ^137^Cs in forests stabilized following the FDNPP accident. These findings are expected to support the validation of models that predict long-term ^137^Cs activity concentrations in stem wood.

## Supplementary Information

Below is the link to the electronic supplementary material.

Supplementary Material 1

Supplementary Material 2

## Data Availability

All data used in this study are available in the Supplementary Information files.
