# Supplementary material for: Decadal stability of radiocesium inventories and soil to tree transfer in forests affected by the Fukushima nuclear accident
Source: Sci Rep. 2026 Jan 7;16:4653. doi: 10.1038/s41598-025-34898-0 (PMC12868700; doi:10.1038/s41598-025-34898-0)
Supplement: Supplementary file 1 — Supplementary Material 1 [file 41598_2025_34898_MOESM1_ESM.docx]

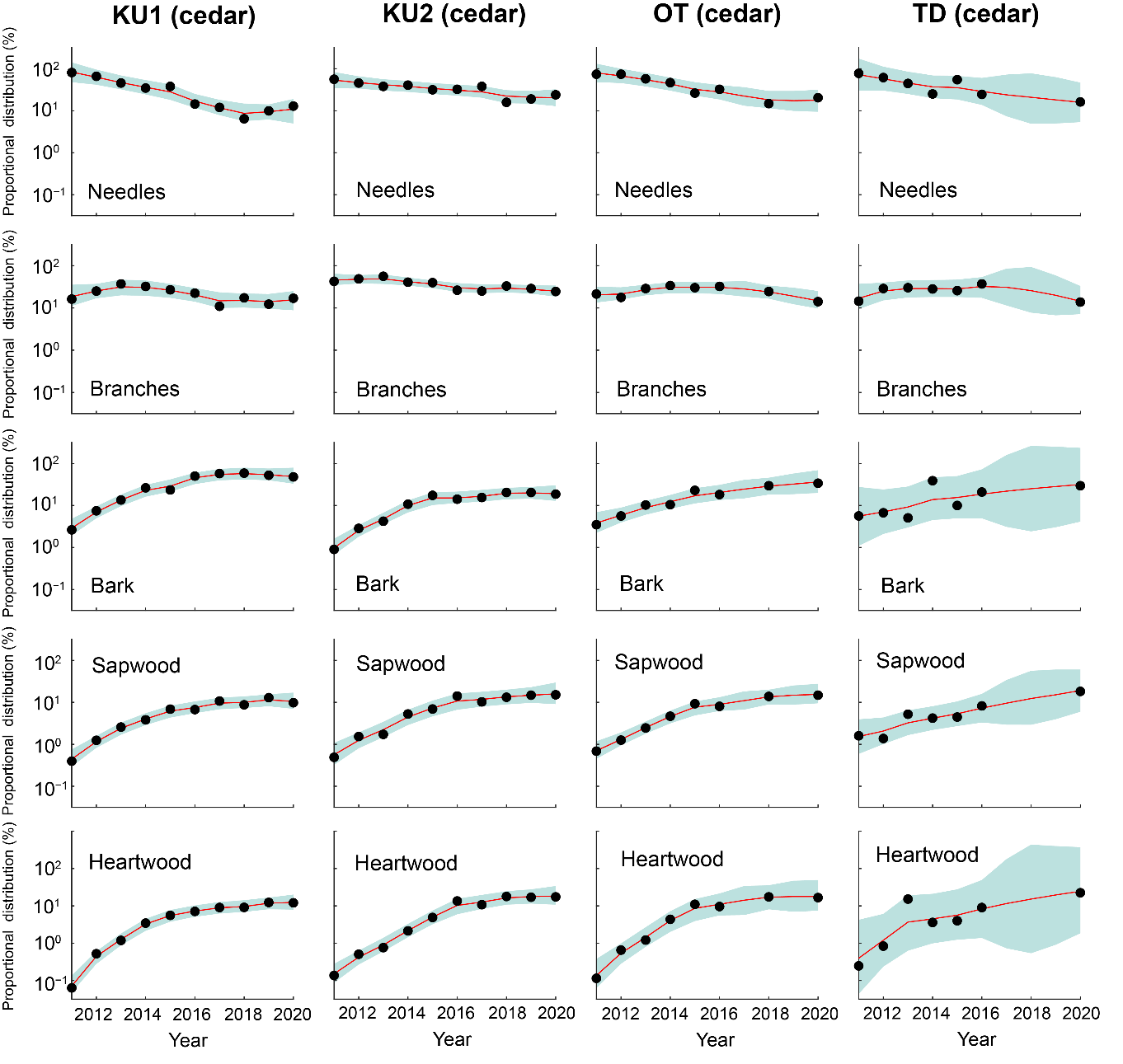


**Figure S1.** Temporal variations in the proportional distribution of ^137^Cs inventory in the aboveground compartments of Japanese cedar at four study sites, fitted using a dynamic linear model (DLM). Black circles represent the observed values, while red lines indicate the DLM fit. Blue shaded areas denote 95% credible intervals.


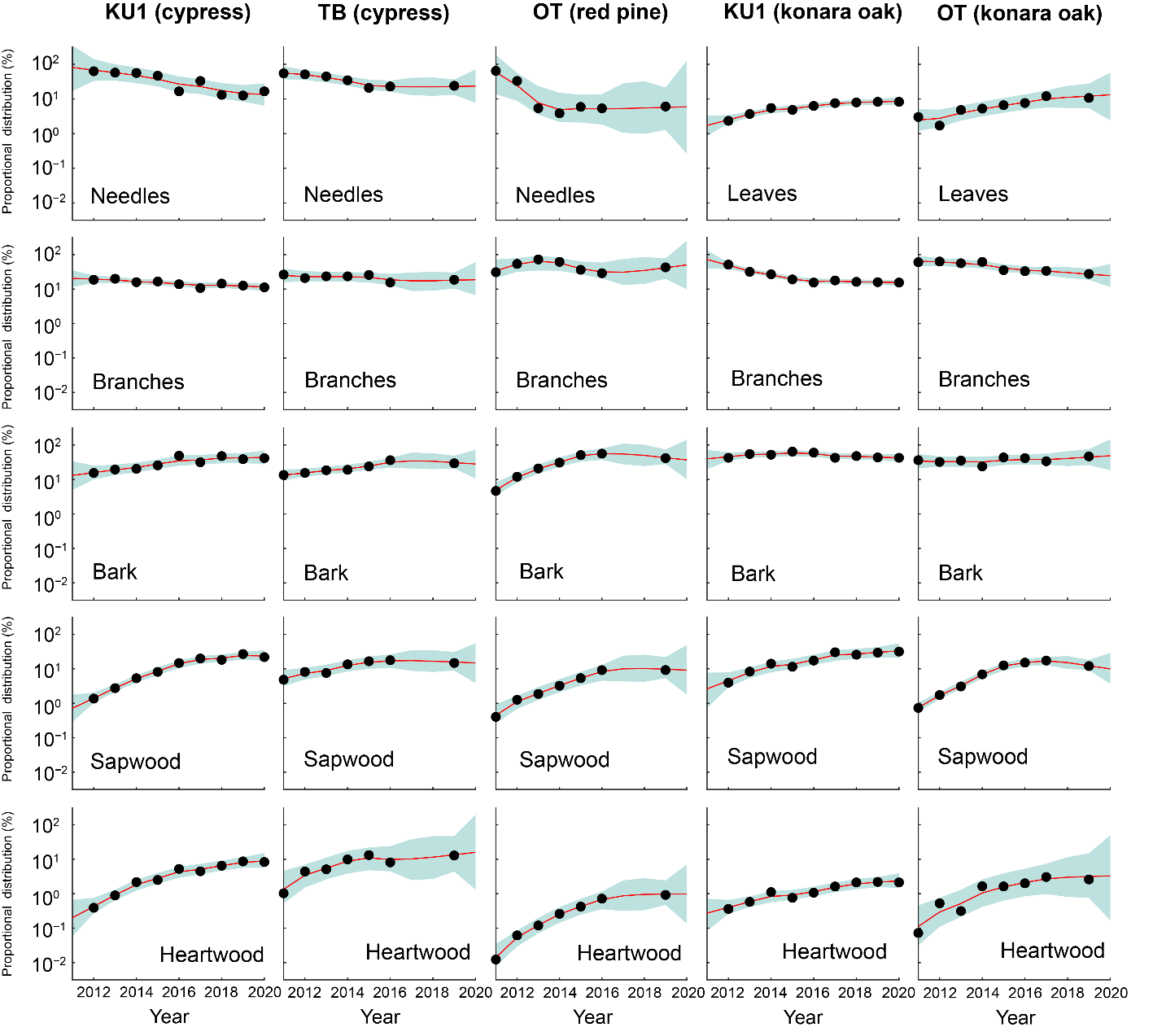


**Figure S2.** Temporal variations in the proportional distribution of ^137^Cs inventory in the aboveground compartments of Japanese cypress, Japanese red pine, and konara oak, fitted using a dynamic linear model (DLM). Black circles represent the observed values, while the red lines indicate the DLM fit. Blue shaded areas denote 95% credible intervals.
